# Crystal structure of 3,14-dimethyl-2,13-di­aza-6,17-diazo­niatri­cyclo­[16.4.0.0^7,12^]docosane bis­(per­chlorate) from synchrotron X-ray data

**DOI:** 10.1107/S2056989021004278

**Published:** 2021-04-23

**Authors:** Dohyun Moon, Sunghwan Jeon, Jong-Ha Choi

**Affiliations:** a Pohang Accelerator Laboratory, POSTECH, Pohang 37673, Republic of Korea; b Department of Chemistry, Andong National University, Andong 36729, Republic of Korea

**Keywords:** crystal structure, protonated macrocycle, perchlorate, hydrogen bonding, synchrotron radiation

## Abstract

The crystal structure of the title salt, C_20_H_42_N_4_^2+^·2ClO_4_^−^, has been determined using synchrotron radiation at 220 (2) K. The structure determination reveals that protonation has occurred at diagonally opposite amine N atoms. The asymmetric unit comprises one half of the organic dication, which lies about a center of inversion, and one perchlorate anion. The macrocyclic dication adopts the most stable endodentate *trans*-III conformation. The crystal structure is stabilized by intra­molecular N—H⋯N, and inter­molecular N—H⋯O and C–H⋯O hydrogen bonds involving the macrocycle N—H and C—H groups as donors and the O atoms of perchlorate anions as acceptors, giving rise to a three-dimensional network.

## Abstract 2

In the title salt, C_20_H_42_N_4_^2+^·2ClO_4_^−^, the macrocyclic dication lies about an inversion center. In the crystal, the organic dication and perchlorate anions are linked through N—H⋯O, C—H⋯O and N—H⋯N hydrogen bonds, forming a three-dimensional network.

## Chemical context   

The macrocyclic compound, 3,14-dimethyl-2,6,13,17-tetra­aza­tri­cyclo­(16.4.0.0^7,12^)docosane (C_20_H_40_N_4_) contains a cyclam backbone with two cyclo­hexane subunits and two methyl groups are also attached to carbon atoms 3 and 14 of the propyl chains that bridge opposite pairs of N atoms in the structure. The macrocycle is basic and readily captures two or four protons to form the [C_20_H_42_N_4_]^2+^ dication or the [C_20_H_44_N_4_]^4+^ tetra­cation in which all of the N—H bonds are generally available for hydrogen-bond formation (Moon *et al.*, 2021 ▸).

Previously, the crystal structures of [Cu(C_20_H_40_N_4_)](NO_3_)_2_·3H_2_O, [Cu(C_20_H_40_N_4_)](NO_3_)_2_, [Cu(C_20_H_40_N_4_)](ClO_4_)_2_ and [Cu(C_20_H_40_N_4_)(H_2_O)_2_](BF_4_)_2_·2H_2_O were reported together with [Zn(C_20_H_40_N_4_)(OCOCH_3_)_2_]. In these structures, the copper(II) or zinc(II) cations have tetra­gonally distorted octa­hedral environments with the four N atoms of the macrocyclic ligand in equatorial positions and the O atoms of the counter-anions, water mol­ecules or acetato ligands in axial positions (Choi *et al.*, 2006 ▸, 2007 ▸, 2012*a* ▸,*b* ▸; Ross *et al.*, 2012 ▸). In these Cu^II^ and Zn^II^ complexes, the macrocyclic ligands adopt their most stable *trans*-III configurations. The crystal structures of (C_20_H_40_N_4_)·2(C_11_H_10_O) (Choi *et al.*, 2012*c* ▸), (C_20_H_40_N_4_)·2(NO_2_OH) (Moon *et al.*, 2020 ▸), [C_20_H_42_N_4_](SO_4_)·2MeOH (White *et al.*, 2015 ▸), [C_20_H_42_N_4_]Br_2_·2H_2_O (Moon *et al.*, 2021 ▸) and [C_20_H_44_N_4_]Br_4_·4H_2_O (Moon *et al.*, 2021 ▸) have also been determined.

We report here the preparation of a new dicationic compound, [C_20_H_42_N_4_](ClO_4_)_2_, (I) and its structural characterization by synchrotron single-crystal X-ray diffraction.
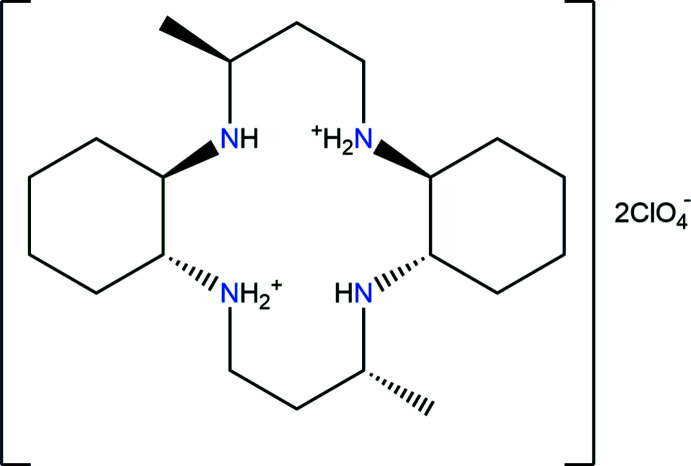


## Structural commentary   

**Figure 1:**
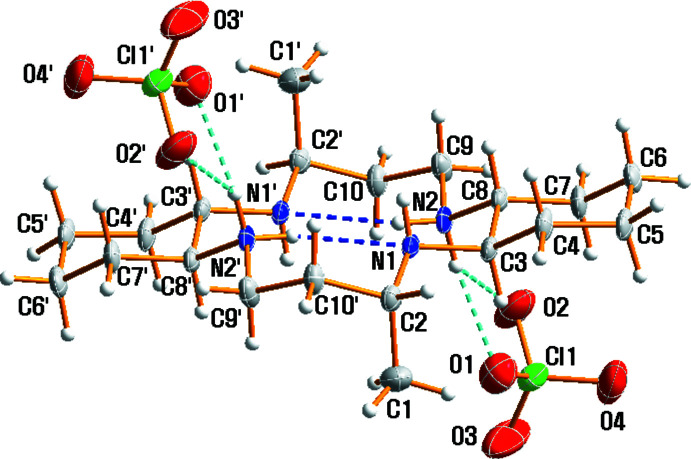
The mol­ecular structure of compound (I), drawn with displacement ellipsoids at the 50% probability level. Dashed lines represent hydrogen-bonding inter­actions and primed atoms are related by the symmetry operation (−*x* + 1, −*y* + 2, −*z* + 1).

An ellipsoid plot of the mol­ecular components in (I) with the atom-numbering scheme is shown in Fig. 1 ▸. The asymmetric unit consists of one half of the macrocyclic dication, which lies about a center of inversion, and one perchlorate anion. The four N atoms are coplanar, and the two methyl substituents are *anti* with respect to the macrocyclic plane as a result of the mol­ecular inversion symmetry. The [C_20_H_42_N_4_]^2+^ dication adopts an endodentate conformation and *trans*-III configuration along the center of the macrocyclic cavity. The endo conformation of the dication may be due to the intra­molecular N—H⋯N hydrogen-bonding inter­action. Within the centrosymmetric diprotonated amine unit, the C—C and N—C bond lengths range from 1.5173 (18) to 1.5368 (18) Å and from 1.4795 (16) to 1.5044 (16) Å, respectively. The range of N—C—C and C—N—C angles is 108.89 (11) to 113.50 (11)° and 113.46 (11) to 114.61 (11)°, respectively. The bond lengths and angles within the dication are comparable to those found in the free ligand or other cations in (C_20_H_40_N_4_)·2C_11_H_10_O (Choi *et al.*, 2012*c* ▸), [C_20_H_42_N_4_](SO_4_)·2MeOH (White *et al.*, 2015 ▸) and [C_20_H_42_N_4_][Fe{HB(pz)_3_}(CN)_3_]_2_·2H_2_O·2MeOH (Kim *et al.*, 2004 ▸; pz = pyrazol­yl). The protonation of the N atoms may depend on the location of the neighboring counter-anions involved in hydrogen bonding. The bond-length difference can be noticed for several N—C bonds. The N—C bond length involving the non-protonated N1 atom is shorter than that involving protonated N2 atom, *e.g*. N1—C2 [1.4817 (18) Å] and N1—C3 [1.4795 (16) Å] are slightly shorter than N2—C8 [1.5044 (16) Å] and N2—C9 [1.4952 (18) Å]. Each of the two hydrogen atoms of N2 and N2′ (−*x* + 1, −*y* + 2, −*z* + 1) is involved in hydrogen bonding with both of the two remaining nitro­gen atoms (Table 1 ▸). The intra­molecular hydrogen bonding plays a substantial role in maintaining the endodentate geometry of the diprotonated macrocyclic cation. The Cl—O bond distances in the tetra­hedral ClO_4_^−^ anion vary from 1.4218 (19) to 1.4529 (16) Å, and the O—Cl—O angles vary from 106.45 (10) to 110.51 (12)°. The distorted geometry of the ClO_4_^−^ anion undoubtedly results from its involvement in hydrogen-bonding inter­actions with the organic cation.

## Supra­molecular features   

**Figure 2:**
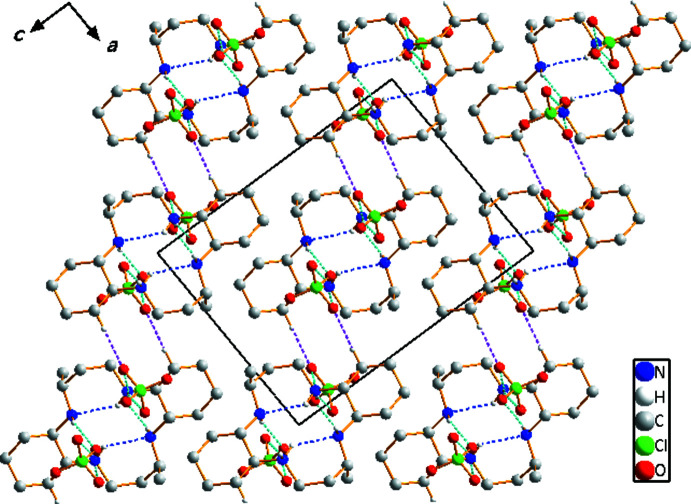
Crystal packing in compound (I), viewed perpendicular to the *ac* plane. Dashed lines represent N—H⋯O (cyan), N—H⋯N (blue) and C—H⋯O (purple) hydrogen-bonding inter­actions, respectively.

Three N—H⋯O, C–H⋯O and N—H⋯N hydrogen-bonding inter­actions occur in the crystal structure (Table 1 ▸). The O atoms of the perchlorate anions serve as hydrogen-bond acceptors. The ClO_4_^−^ anions are connected to the [C_20_H_42_N_4_]^2+^ dication by N—H⋯O hydrogen bonds. The macrocyclic dication is linked to a neighboring ClO_4_^−^ anion through a very weak C—H⋯O hydrogen bond. The extensive array of these contacts generates a three-dimensional network structure (Fig. 2 ▸), and these hydrogen-bonding inter­actions help to stabilize the crystal structure.

## Database survey   

A search of the Cambridge Structural (Version 5.42, Update 1, February 2021; Groom *et al.*, 2016 ▸) indicated 121 hits for organic and transition-metal compounds containing the macrocycles (C_20_H_40_N_4_), [C_20_H_42_N_4_]^2+^ or [C_20_H_44_N_4_]^4+^. The crystal structures of (C_20_H_40_N_4_)·2C_11_H_10_O (Choi *et al.*, 2012*c* ▸), [C_20_H_42_N_4_](SO_4_)·2MeOH (White *et al.*, 2015 ▸), [C_20_H_42_N_4_]Br_2_·2H_2_O (Moon *et al.*, 2021 ▸), [C_20_H_44_N_4_]Cl_4_·4H_2_O (Moon *et al.*, 2018 ▸) and [C_20_H_44_N_4_]Br_4_·4H_2_O (Moon *et al.*, 2021 ▸) were reported previously and commented on in the *Chemical context* section.

## Synthesis and crystallization   

Commercially available *trans*-1,2-cyclo­hexa­nedi­amine and methyl vinyl ketone (Sigma-Aldrich) were used as provided. All chemicals were reagent grade and used without further purification. As a starting material, macrocycle 3,14-dimethyl-2,6,13,17-tetra­aza­tri­cyclo­(16.4.0.0^7,12^)docosane, *L*, was prepared according to a published procedure (Kang *et al.*, 1991 ▸). Macrocycle *L* (0.034 g, 0.1 mmol) was suspended in methanol (20 mL) and the pH was adjusted to 3.0 with 0.5 *M* HClO_4_. The mixture was stirred magnetically for 30 min and the resulting solution was filtered. The neat filtrate was allowed to stand for one week to give block-like colorless crystals of (I) suitable for X-ray structural analysis.

## Refinement   

Crystal data, data collection and structure refinement details are summarized in Table 2 ▸. All non-hydrogen atoms were refined anisotropically. All C-bound H atoms and the hydrogen atoms of the diprotonated amine (H2*A* and H2*B*) were placed in geometrically idealized positions and constrained to ride on their parent atoms, with C—H distances of 0.97–0.98 Å and an N—H distance of 0.99 Å, and with *U*_iso_(H) values of 1.5 and 1.2 times, respectively, that of the parent atoms. The one N-bound H atom (H1*N*1) of the amine was assigned based on a difference-Fourier map, and a *U*_iso_(H) value of 1.5*U*_eq_(N1).

## Supplementary Material

Crystal structure: contains datablock(s) I. DOI: 10.1107/S2056989021004278/vm2247sup1.cif

Structure factors: contains datablock(s) I. DOI: 10.1107/S2056989021004278/vm2247Isup2.hkl

Supporting information file. DOI: 10.1107/S2056989021004278/vm2247Isup3.cml

CCDC reference: 2079010

Additional supporting information:  crystallographic information; 3D view; checkCIF report

## Figures and Tables

**Table 1:** Hydrogen-bond geometry (Å, °)

*D*—H⋯*A*	*D*—H	H⋯*A*	*D*⋯*A*	*D*—H⋯*A*
N1—H1*N*1⋯O3^i^	0.86 (2)	2.22 (2)	3.007 (2)	152.4 (18)
N2—H2*A*⋯O1	0.90	2.09	2.970 (2)	164
N2—H2*A*⋯O2	0.90	2.56	3.239 (2)	132
N2—H2*B*⋯N1^ii^	0.90	2.29	2.9846 (16)	134
N2—H2*B*⋯N1	0.90	2.39	2.8230 (17)	109
C7—H7*A*⋯O2^iii^	0.98	2.57	3.423 (3)	145

Symmetry codes: (i) x, y+1, z; (ii) -x+1, -y+2, -z+1; (iii) -x+{\script{3\over 2}}, y+{\script{1\over 2}}, -z+{\script{3\over 2}}.

**Table 2:** Experimental details

Crystal data	
Chemical formula	C_20_H_42_N_4_^2+^·2ClO_4_^−^
*M*_r_	537.47
Crystal system, space group	Monoclinic, *P*2_1_/*n*
Temperature (K)	220
*a*, *b*, *c* (Å)	10.689 (2), 8.4450 (17), 14.020 (3)
β (°)	92.90 (3)
*V* (Å^3^)	1263.9 (4)
*Z*	2
Radiation type	Synchrotron, λ = 0.630 Å
μ (mm^−1^)	0.22
Crystal size (mm)	0.08 × 0.08 × 0.08

Data collection	
Diffractometer	Rayonix MX225HS CCD area detector
Absorption correction	Empirical (using intensity measurements) (*HKL3000sm* *SCALEPACK*; Otwinowski *et al.*, 2003 ▸)
*T*_min_, *T*_max_	0.957, 1.000
No. of measured, independent and observed [*I* > 2σ(*I*)] reflections	12842, 3549, 3164
*R*_int_	0.063
(sin θ/λ)_max_ (Å^−1^)	0.696

Refinement	
*R*[*F* ^2^ > 2σ(*F* ^2^)], *wR*(*F* ^2^), *S*	0.055, 0.172, 1.11
No. of reflections	3549
No. of parameters	158
H-atom treatment	H atoms treated by a mixture of independent and constrained refinement
Δρ_max_, Δρ_min_ (e Å^−3^)	0.86, −0.44

Computer programs: *PAL BL2D-SMDC* (Shin *et al.*, 2016 ▸), *HKL3000sm* (Otwinowski & Minor, 1997 ▸), *SHELXT2018* (Sheldrick, 2015*a* ▸), *SHELXL2018* (Sheldrick, 2015*b* ▸), *DIAMOND 4* (Putz & Brandenburg, 2014 ▸) and *publCIF* (Westrip, 2010 ▸).

## supplementary crystallographic information

### Crystal data

**Table:**

C_20_H_42_N_4_^2+^·2ClO_4_^−^	*F*(000) = 576
*M**_r_* = 537.47	*D*_x_ = 1.412 Mg m^−^^3^
Monoclinic, *P*2_1_/*n*	Synchrotron radiation, λ = 0.630 Å
*a* = 10.689 (2) Å	Cell parameters from 41946 reflections
*b* = 8.4450 (17) Å	θ = 0.4–33.6°
*c* = 14.020 (3) Å	µ = 0.22 mm^−^^1^
β = 92.90 (3)°	*T* = 220 K
*V* = 1263.9 (4) Å^3^	Block, colorless
*Z* = 2	0.08 × 0.08 × 0.08 mm

### Data collection

**Table:**

Rayonix MX225HS CCD area detector diffractometer	3164 reflections with *I* > 2σ(*I*)
Radiation source: PLSII 2D bending magnet	*R*_int_ = 0.063
ω scan	θ_max_ = 26.0°, θ_min_ = 2.5°
Absorption correction: empirical (using intensity measurements) (*HKL3000sm* Scalepack; Otwinowski *et al.*, 2003)	*h* = −14→14
*T*_min_ = 0.957, *T*_max_ = 1.000	*k* = −11→11
12842 measured reflections	*l* = −19→19
3549 independent reflections	

### Refinement

**Table:**

Refinement on *F*^2^	0 restraints
Least-squares matrix: full	Hydrogen site location: mixed
*R*[*F*^2^ > 2σ(*F*^2^)] = 0.055	H atoms treated by a mixture of independent and constrained refinement
*wR*(*F*^2^) = 0.172	*w* = 1/[σ^2^(*F*_o_^2^) + (0.1016*P*)^2^ + 0.4023*P*] where *P* = (*F*_o_^2^ + 2*F*_c_^2^)/3
*S* = 1.11	(Δ/σ)_max_ < 0.001
3549 reflections	Δρ_max_ = 0.86 e Å^−^^3^
158 parameters	Δρ_min_ = −0.44 e Å^−^^3^

### Special details

**Table:**

Geometry. All esds (except the esd in the dihedral angle between two l.s. planes) are estimated using the full covariance matrix. The cell esds are taken into account individually in the estimation of esds in distances, angles and torsion angles; correlations between esds in cell parameters are only used when they are defined by crystal symmetry. An approximate (isotropic) treatment of cell esds is used for estimating esds involving l.s. planes.

### Fractional atomic coordinates and isotropic or equivalent isotropic displacement parameters (Å^2^)

**Table:**

	*x*	*y*	*z*	*U*_iso_*/*U*_eq_	
N1	0.35546 (11)	1.04262 (14)	0.58019 (8)	0.0179 (2)	
H1N1	0.3881 (18)	1.135 (3)	0.5913 (14)	0.027*	
N2	0.58037 (10)	0.87373 (14)	0.61832 (7)	0.0189 (2)	
H2A	0.556980	0.771653	0.612584	0.023*	
H2B	0.556374	0.923391	0.563613	0.023*	
C1	0.17869 (17)	0.8824 (2)	0.51236 (13)	0.0357 (4)	
H1A	0.104402	0.890989	0.470004	0.054*	
H1B	0.158772	0.823502	0.569045	0.054*	
H1C	0.243896	0.827482	0.479816	0.054*	
C2	0.22417 (13)	1.04715 (17)	0.54095 (10)	0.0208 (3)	
H2	0.171026	1.086474	0.591704	0.025*	
C3	0.37355 (13)	0.95865 (16)	0.67248 (9)	0.0186 (3)	
H3	0.339745	0.850008	0.664720	0.022*	
C4	0.30834 (15)	1.03951 (18)	0.75469 (10)	0.0254 (3)	
H4A	0.337406	1.149317	0.760396	0.030*	
H4B	0.217795	1.041505	0.739748	0.030*	
C5	0.33416 (16)	0.95505 (19)	0.85002 (10)	0.0270 (3)	
H5A	0.296287	1.015186	0.900932	0.032*	
H5B	0.295502	0.849816	0.847313	0.032*	
C6	0.47429 (16)	0.9383 (2)	0.87300 (10)	0.0284 (3)	
H6A	0.488076	0.877269	0.932053	0.034*	
H6B	0.511244	1.043474	0.883362	0.034*	
C7	0.53896 (14)	0.85522 (19)	0.79198 (9)	0.0258 (3)	
H7A	0.629365	0.849658	0.807106	0.031*	
H7B	0.506976	0.746930	0.784522	0.031*	
C8	0.51363 (13)	0.94746 (16)	0.69916 (9)	0.0187 (3)	
H8	0.546260	1.056300	0.708844	0.022*	
C9	0.71994 (13)	0.8814 (2)	0.63136 (9)	0.0257 (3)	
H9A	0.747588	0.810782	0.683724	0.031*	
H9B	0.744648	0.989510	0.649456	0.031*	
C10	0.78531 (13)	0.83475 (19)	0.54199 (9)	0.0239 (3)	
H10A	0.750585	0.733364	0.519265	0.029*	
H10B	0.874193	0.817560	0.559494	0.029*	
Cl1	0.54918 (4)	0.43816 (5)	0.65415 (3)	0.03296 (16)	
O1	0.45977 (15)	0.55690 (19)	0.61926 (13)	0.0495 (4)	
O2	0.67029 (15)	0.5118 (2)	0.65563 (14)	0.0618 (5)	
O3	0.5462 (2)	0.3046 (2)	0.59233 (17)	0.0762 (6)	
O4	0.51936 (16)	0.3923 (2)	0.74864 (12)	0.0598 (5)	

### Atomic displacement parameters (Å^2^)

**Table:**

	*U*^11^	*U*^22^	*U*^33^	*U*^12^	*U*^13^	*U*^23^
N1	0.0238 (5)	0.0176 (5)	0.0127 (5)	−0.0015 (4)	0.0047 (4)	0.0024 (4)
N2	0.0246 (5)	0.0218 (6)	0.0109 (4)	0.0014 (4)	0.0056 (4)	0.0023 (4)
C1	0.0388 (8)	0.0307 (8)	0.0374 (9)	−0.0110 (7)	−0.0011 (6)	−0.0010 (7)
C2	0.0229 (6)	0.0235 (7)	0.0165 (6)	0.0007 (5)	0.0065 (4)	0.0003 (5)
C3	0.0262 (6)	0.0175 (6)	0.0128 (5)	0.0000 (5)	0.0073 (4)	0.0028 (4)
C4	0.0347 (7)	0.0271 (7)	0.0155 (6)	0.0061 (6)	0.0121 (5)	0.0040 (5)
C5	0.0407 (8)	0.0275 (7)	0.0139 (6)	0.0017 (6)	0.0128 (5)	0.0031 (5)
C6	0.0427 (8)	0.0326 (8)	0.0105 (6)	−0.0001 (6)	0.0061 (5)	0.0000 (5)
C7	0.0349 (7)	0.0315 (7)	0.0113 (5)	0.0049 (6)	0.0060 (5)	0.0046 (5)
C8	0.0265 (6)	0.0194 (6)	0.0106 (5)	−0.0004 (5)	0.0063 (4)	0.0006 (4)
C9	0.0242 (6)	0.0387 (8)	0.0146 (6)	0.0006 (6)	0.0047 (4)	0.0006 (5)
C10	0.0264 (6)	0.0291 (7)	0.0168 (6)	0.0062 (5)	0.0063 (5)	0.0030 (5)
Cl1	0.0328 (2)	0.0270 (3)	0.0393 (3)	−0.00471 (14)	0.00435 (17)	0.00776 (14)
O1	0.0454 (8)	0.0462 (9)	0.0564 (9)	0.0068 (6)	−0.0015 (7)	0.0120 (7)
O2	0.0423 (8)	0.0658 (11)	0.0770 (12)	−0.0215 (8)	0.0003 (8)	0.0287 (10)
O3	0.1111 (16)	0.0318 (8)	0.0893 (14)	−0.0193 (10)	0.0399 (12)	−0.0118 (9)
O4	0.0577 (9)	0.0755 (12)	0.0463 (9)	−0.0126 (9)	0.0036 (7)	0.0284 (9)

### Geometric parameters (Å, º)

**Table:**

N1—C3	1.4795 (16)	C5—C6	1.523 (2)
N1—C2	1.4817 (18)	C5—H5A	0.9800
N1—H1N1	0.86 (2)	C5—H5B	0.9800
N2—C9	1.4952 (18)	C6—C7	1.530 (2)
N2—C8	1.5044 (16)	C6—H6A	0.9800
N2—H2A	0.9000	C6—H6B	0.9800
N2—H2B	0.9000	C7—C8	1.5290 (18)
C1—C2	1.521 (2)	C7—H7A	0.9800
C1—H1A	0.9700	C7—H7B	0.9800
C1—H1B	0.9700	C8—H8	0.9900
C1—H1C	0.9700	C9—C10	1.5173 (18)
C2—C10^i^	1.5314 (19)	C9—H9A	0.9800
C2—H2	0.9900	C9—H9B	0.9800
C3—C8	1.5278 (19)	C10—H10A	0.9800
C3—C4	1.5368 (18)	C10—H10B	0.9800
C3—H3	0.9900	Cl1—O3	1.4218 (19)
C4—C5	1.528 (2)	Cl1—O4	1.4315 (16)
C4—H4A	0.9800	Cl1—O2	1.4354 (15)
C4—H4B	0.9800	Cl1—O1	1.4529 (16)
			
C3—N1—C2	114.61 (11)	H5A—C5—H5B	108.0
C3—N1—H1N1	103.8 (13)	C5—C6—C7	111.23 (13)
C2—N1—H1N1	114.3 (13)	C5—C6—H6A	109.4
C9—N2—C8	113.46 (11)	C7—C6—H6A	109.4
C9—N2—H2A	108.9	C5—C6—H6B	109.4
C8—N2—H2A	108.9	C7—C6—H6B	109.4
C9—N2—H2B	108.9	H6A—C6—H6B	108.0
C8—N2—H2B	108.9	C8—C7—C6	109.34 (13)
H2A—N2—H2B	107.7	C8—C7—H7A	109.8
C2—C1—H1A	109.5	C6—C7—H7A	109.8
C2—C1—H1B	109.5	C8—C7—H7B	109.8
H1A—C1—H1B	109.5	C6—C7—H7B	109.8
C2—C1—H1C	109.5	H7A—C7—H7B	108.3
H1A—C1—H1C	109.5	N2—C8—C3	109.71 (11)
H1B—C1—H1C	109.5	N2—C8—C7	111.10 (11)
N1—C2—C1	110.99 (12)	C3—C8—C7	111.66 (11)
N1—C2—C10^i^	108.89 (11)	N2—C8—H8	108.1
C1—C2—C10^i^	112.81 (13)	C3—C8—H8	108.1
N1—C2—H2	108.0	C7—C8—H8	108.1
C1—C2—H2	108.0	N2—C9—C10	112.70 (11)
C10^i^—C2—H2	108.0	N2—C9—H9A	109.1
N1—C3—C8	109.08 (10)	C10—C9—H9A	109.1
N1—C3—C4	113.50 (11)	N2—C9—H9B	109.1
C8—C3—C4	108.65 (12)	C10—C9—H9B	109.1
N1—C3—H3	108.5	H9A—C9—H9B	107.8
C8—C3—H3	108.5	C9—C10—C2^i^	116.25 (13)
C4—C3—H3	108.5	C9—C10—H10A	108.2
C5—C4—C3	112.33 (12)	C2^i^—C10—H10A	108.2
C5—C4—H4A	109.1	C9—C10—H10B	108.2
C3—C4—H4A	109.1	C2^i^—C10—H10B	108.2
C5—C4—H4B	109.1	H10A—C10—H10B	107.4
C3—C4—H4B	109.1	O3—Cl1—O4	110.51 (12)
H4A—C4—H4B	107.9	O3—Cl1—O2	110.20 (14)
C6—C5—C4	111.15 (12)	O4—Cl1—O2	110.28 (11)
C6—C5—H5A	109.4	O3—Cl1—O1	110.37 (13)
C4—C5—H5A	109.4	O4—Cl1—O1	108.95 (11)
C6—C5—H5B	109.4	O2—Cl1—O1	106.45 (10)
C4—C5—H5B	109.4		
			
C3—N1—C2—C1	−67.02 (15)	C9—N2—C8—C7	−65.28 (15)
C3—N1—C2—C10^i^	168.21 (11)	N1—C3—C8—N2	−53.86 (14)
C2—N1—C3—C8	173.99 (10)	C4—C3—C8—N2	−178.05 (10)
C2—N1—C3—C4	−64.72 (15)	N1—C3—C8—C7	−177.48 (11)
N1—C3—C4—C5	−177.06 (12)	C4—C3—C8—C7	58.32 (15)
C8—C3—C4—C5	−55.52 (16)	C6—C7—C8—N2	177.49 (12)
C3—C4—C5—C6	54.72 (17)	C6—C7—C8—C3	−59.68 (16)
C4—C5—C6—C7	−55.07 (17)	C8—N2—C9—C10	−169.40 (12)
C5—C6—C7—C8	57.14 (16)	N2—C9—C10—C2^i^	71.50 (17)
C9—N2—C8—C3	170.77 (11)		

Symmetry code: (i) −*x*+1, −*y*+2, −*z*+1.

### Hydrogen-bond geometry (Å, º)

**Table:**

*D*—H···*A*	*D*—H	H···*A*	*D*···*A*	*D*—H···*A*
N1—H1*N*1···O3^ii^	0.86 (2)	2.22 (2)	3.007 (2)	152.4 (18)
N2—H2*A*···O1	0.90	2.09	2.970 (2)	164
N2—H2*A*···O2	0.90	2.56	3.239 (2)	132
N2—H2*B*···N1^i^	0.90	2.29	2.9846 (16)	134
N2—H2*B*···N1	0.90	2.39	2.8230 (17)	109
C7—H7*A*···O2^iii^	0.98	2.57	3.423 (3)	145

Symmetry codes: (i) −*x*+1, −*y*+2, −*z*+1; (ii) *x*, *y*+1, *z*; (iii) −*x*+3/2, *y*+1/2, −*z*+3/2.
